# Barriers and facilitators of benzathine penicillin G adherence among rheumatic heart disease patients: a mixed methods systematic review using the COM-B (capability, opportunity, and motivation for behavior) model

**DOI:** 10.1186/s13643-024-02691-1

**Published:** 2024-12-03

**Authors:** Habtamu Abera Areri, Henok Tadele, Sale Workneh

**Affiliations:** 1 School of Nursing and Midwifery, College of Health Sciences, Addis Ababa University, Addis Ababa, Ethiopia; 2 Department of Pediatrics and Child Health, School of Medicine, College of Health Sciences, Addis Ababa University, Addis Ababa, Ethiopia; 3 School of Public Health, College of Health Sciences, Addis Ababa University, Addis Ababa, Ethiopia

**Keywords:** Barriers, Facilitators, Benzathine Penicillin G, Rheumatic heart disease, Adherence

## Abstract

**Background:**

Benzathine penicillin G (BPG) is a proven preventive agent for preventing the progression of rheumatic heart disease (RHD) and is recognized as a standard of care. However, ensuring adherence to BPG remains a global challenge. The objective of this review was to synthesize the available evidence on the barriers to and facilitators of BPG adherence among RHD patients.

**Methods:**

This systematic review included both qualitative and quantitative studies on RHD patients published in the English language. This systematic review was conducted following the Preferred Reporting Items for Systematic Reviews and Meta-analyses (PRISMA) guidelines. The search strategy involved PubMed, Embase, CINAHL, Global Health, Scopus, and Web of Sciences databases to identify keywords and terms contained in the title and abstract and the index terms used to describe articles. The review included papers published from January 1, 2000, to March 30, 2024. Two independent reviewers screened, appraised, and extracted the data. The data analysis was carried out deductively to fit onto the components of the COM-B (Capability, Opportunity, Motivation-Behaviour) model.

**Results:**

In this review, 1067 records were screened, and 22 studies with 7338 participants were included. Thirty-five barriers and twenty facilitators were identified and mapped onto COM-B components. Physical capability (e.g., felt healthy), psychological capability (e.g., lack of knowledge), reflective motivation (e.g., poor patient handling), automatic motivation (e.g., BPG injection pain), physical opportunity (e.g., BPG unavailability) and social opportunity (e.g., inadequate counseling) were identified as barriers. The most discussed barrier was automatic motivation, followed by psychological capability and physical opportunity.

**Conclusions:**

Our review revealed variable levels of BPG adherence across studies and identified significant barriers and facilitators. Further research is recommended to identify contextual interventions to address barriers and capitalize on facilitators.

**Systematic review registration:**

PROSPERO CRD42024535398.

**Supplementary Information:**

The online version contains supplementary material available at 10.1186/s13643-024-02691-1.

## Background

Globally, more than 40 million individuals were affected by rheumatic heart disease (RHD) in 2019, resulting in more than 10 million disability-adjusted life years and more than 306,000 deaths [1]. In Central and South Asia, the Middle East, the Pacific, Sub-Saharan Africa, and older adults in high-income countries, the prevalence of RHD remains high [2]. In sub-Saharan Africa, including Ethiopia, the prevalence of RHD is among the highest in the world, and its prevalence remains high in people with poor living conditions and limited healthcare access [2, 3].

Secondary prophylaxis with benzathine penicillin G (BPG) is a proven preventive strategy for preventing RHD progression and is recognized as a standard of care. Every 4 weeks or 28 days, intramuscular BPG injection is the preferred dosing for preventing the progression of established RHD by many technical experts, as well as averting morbidity and mortality [4]. However, continuous protection from recurrent rheumatic fever requires an optimal level of BPG prophylaxis adherence, but maintaining high adherence has always remained a global challenge, with a pooled prevalence of BPG adherence of 46% [4, 5]. Ensuring adherence to prophylaxis has proven to be a global challenge for various reasons related to patients, healthcare providers, and healthcare systems. For instance, fear of an allergic reaction to benzathine penicillin remains a key concern for healthcare providers [6]. Patient-related factors such as limited healthcare access, living in rural areas, distance from healthcare facilities, poor communication between patients and healthcare providers, and fear of pain are barriers to BPG prophylaxis adherence [7–10]. A lack of family support, conscious refusal, and a lack of reminders were found to be additional patient-related barriers [10]. Healthcare providers’ factors, such as inadequate knowledge of healthcare providers to diagnose and manage RHD-related conditions [2], shortage of BPG [2, 7, 10], inadequate availability of staff, negative perceptions of secondary prophylaxis [10], and inadequate counseling and distance [7], were identified as barriers.

On the other hand, the literature has also identified facilitators related to patients, healthcare communication, and social environments. Patient-related factors such as confidence in the healthcare system [10], fear of previous symptoms of acute rheumatic fever (ARF) [11] or worsening of RHD while missing BPG, personal motivation [8], higher educational status [12], higher treatment costs, and better RHD knowledge [9] were found to be facilitators of BPG adherence. Healthcare-related factors such as a recall/reminder system, appropriately trained [10] and dedicated health teams for BPG services [13], patient education about RHD, a community support system or community-based service delivery [13], accessible healthcare, home visits [14], and a secure drug supply [2] were identified as facilitators. A positive and strong therapeutic interaction between patients and healthcare staff is also the most common facilitator [10, 15]. Finally, support from family/friends was found to facilitate better BPG adherence [8, 9, 14].

**Fig. 1:**
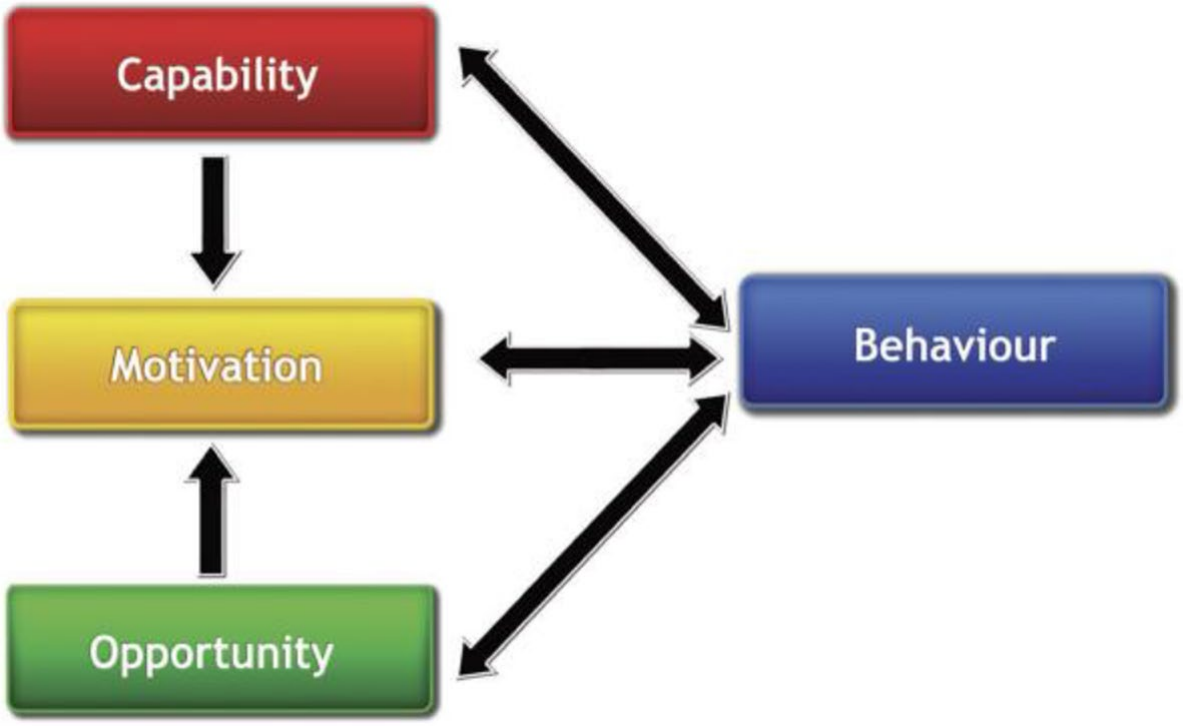
COM-B model [24]

**Fig. 2:**
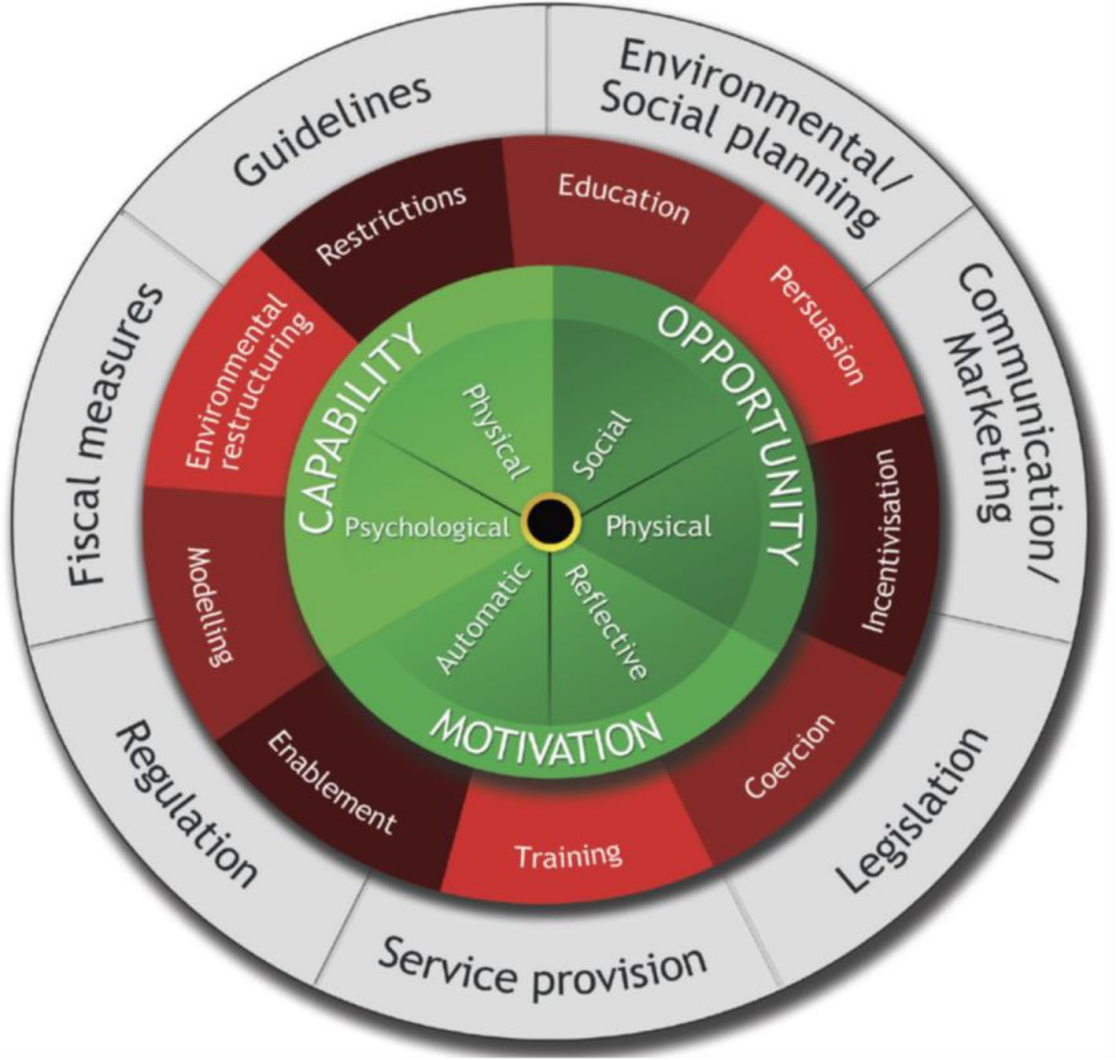
Behavioral change wheel [16]

It is essential to further identify barriers and capitalize on enabling factors from globally available data and then explain the findings using the behavioral change model. The COM-B model (capability, opportunity, motivation-behavior) is commonly used in behavior change studies, and it best explains the factors and identifies interventions using the Behavior Change Wheel (BCW). Capability (e.g., knowledge), opportunity (e.g., resources), and motivation (e.g., beliefs) can either facilitate or prevent behavioral change [16] (see Figs. 1 and 2). The COM-B model, which is widely used for the synthesis of evidence in a systematic review of barriers and facilitators [17–23], was used to map our review findings onto its components. The BCW and the Behavior Change Technique Taxonomy facilitated our selection of intervention strategies to address the barriers and facilitators mapped onto each COM-B component. The Behavioral Change Technique helped us identify the content and approach of the intervention linked to the identified behavior. In other words, how the intervention functions are delivered can be described using the Taxonomy of Behavioral Change Techniques [16, 24]. To the best of our knowledge, there has been no systematic review on BPG prophylaxis adherence among confirmed RHD patients using the COM-B model. Therefore, this review aimed to synthesize the available evidence on the barriers to and facilitators of BPG adherence among RHD patients using the COM-B framework and to identify intervention strategies to improve BPG adherence using the BCW.

## Methods

### Protocol registrations

The proposed systematic review was conducted in accordance with the methodology of the Joanna Briggs Institute (JBI) for mixed methods systematic review (MMSR) [25]. This review was conducted as per previously determined inclusion and exclusion criteria in our registered protocol on PROSPERO [registration number: CRD42024535398], available from:

https://www.crd.york.ac.uk/prospero/display_record.php?ID=CRD42024535398 [26]

#### Search strategy

The search strategy used the PICOs format (population, intervention, comparison, outcomes, and study design) to locate peer-reviewed published studies in the English language from January 1, 2000, to March 30, 2024. An initial limited search of PubMed and CINAHL was undertaken to identify keywords and text words contained in the articles on the topic. The text words contained in the titles and abstracts of relevant articles and the index terms used to describe the articles were used to develop a full search strategy for the PubMed, Embase, CINAHL, Global Health, Scopus, and Web of Sciences databases. The search strategy, including all identified keywords and index terms, was adapted for each included database and/or information source. The reference lists of all included sources of evidence were screened for additional studies (Supplementary material 1).

### Eligibility criteria

#### Population

This review included primary studies published with reports of adherence to BPG prophylaxis among confirmed RHD patients of all ages or discussing barriers and facilitators of BPG adherence. The review included studies from both developed and developing countries. The review excluded studies involving symptomatic treatment of ARF, oral antibiotic regimens prescribed as secondary prophylaxis for RHD patients, and guidelines.

### Intervention

The review included studies with RHD patients taking intramuscular injections of BPG but excluded RHD patients on oral drugs for the prevention of RHD progression.

### Outcomes

To minimize the risk of exclusion of important variables, the review considered studies with primary or secondary outcomes of barriers and facilitators to BPG injection adherence.

### Study

Both quantitative and qualitative studies, such as cross-sectional studies (13), longitudinal (5), and qualitative studies (4), whose full texts were available were included in the review.

#### Study selection

**Fig. 3:**
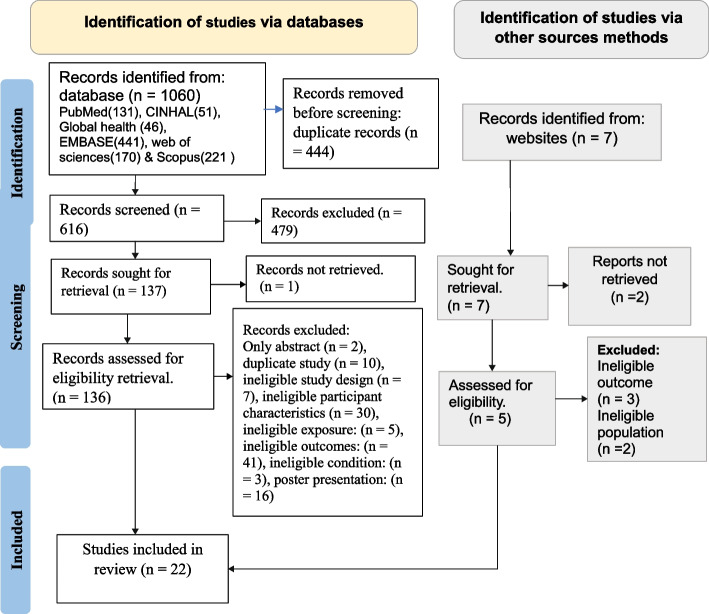
PRISMA flow diagram of the search and study selection process [28]

Following the search, all identified citations were collated and uploaded into EndNote version 21.0, and duplicates were removed. Two independent reviewers (SW and HT) screened the titles and abstracts according to the eligibility criteria. Any disagreement for selection during screening was resolved through discussion and/or by discussion with a third reviewer (HA). Potentially relevant studies were retrieved in full, and their citation details were imported into the JBI System for the Unified Management, Assessment, and Review of Information (JBI SUMARI) (JBI, Adelaide, Australia) [27]. The full texts of the selected studies were assessed in detail against the inclusion criteria by two independent reviewers (HT and SW). The results of the search, reason for exclusion, and inclusion process are reported in full in the final systematic review and presented in a Preferred Reporting Items for Systematic Reviews and Meta-analyses (PRISMA) flow diagram [28] (see Fig. 3)

### Assessment of methodological quality

Two independent reviewers appraised all included studies (HT and SW). Quantitative papers (and a quantitative component of mixed methods papers) were selected for retrieval and assessed by two independent reviewers at the study level for methodological validity before inclusion in the review using standardized critical appraisal instruments from JBI [29]. Qualitative papers (and qualitative components of mixed methods papers) were selected for retrieval and assessed by two independent reviewers for methodological validity prior to inclusion in the review using the standardized critical appraisal instrument from JBI [30]. Studies scoring ≥ 80% out of the maximum number were considered to have strong methodological quality, studies scoring 50–80% were considered to have moderate methodological quality, and studies scoring less than 50% were considered to have low methodological quality. Disagreements were resolved through discussion and supported by the literature [31]. All studies scored 50% or more on the critical appraisal items, which means that all included studies had moderate methodological quality (Supplementary material 2).

### Data extraction and data transformation

The data were extracted by two independent reviewers (HT and HA) using the standardized JBI data extraction tool in JBI SUMARI [32]. The JBI SUMARI data extraction platform included detailed information about the study population, methodology, and outcomes. The quantitative data included descriptive and/or inferential statistical data. Qualitative data is composed of themes or subthemes with corresponding illustrations assigned a level of credibility [27] (Supplementary material 3: Table S3). Any disagreements that were raised between the reviewers were resolved through discussion or with a third reviewer (SW).

Following extraction, quantitative data were transformed into qualitative data (qualitized) to facilitate integration with data extracted from qualitative studies (and qualitative components of mixed methods studies). The “qualitized data” approach involves the transformation of quantitative data into textual descriptions or narrative interpretations to respond directly to the review question. The qualitized data comprised a sample, textual description of descriptive and inferential statistics using average or percentage scores, and declarative stand-alone sentences in a way that answers the review question (Supplementary material 4: Table S2). These textual descriptions are then assembled and pooled with qualitative data extracted directly from qualitative studies.

## Data synthesis and integration

To answer our review question on the identification of barriers and facilitators of BPG adherence among RHD patients, a convergent integrated approach was used to synthesize the data and integrate the findings according to the JBI methodology for Mixed Methods Systematic Review using JBI SUMARI [25, 32]. Synthesis and integration involved assembling the “qualitized” data with the qualitative data. Assembled data were categorized and pooled together based on similarity in meaning to produce a set of integrated findings in the form of line of action statements which were aligned to the review question. The integrated findings were configured under COM-B model components (Supplementary material 4:Table S4). The barriers and facilitators of BPG adherence were then mapped into the six subcomponents of the COM-B model (psychological capability, physical capability, social opportunity, physical opportunity, automatic motivation, and reflective motivation) by two independent reviewers (HA and HT). Disagreements were resolved through discussion with a third reviewer (SW).

## Results

### Study inclusion

Figure 3 shows the PRISMA flowchart search and review process for study selection and inclusion [28]. Our review identified 1067 records, 116 of which were found to be eligible for full-text screening, and 22 of these papers met the inclusion criteria. The review included 18 quantitative and 4 qualitative studies with a total of 7338 RHD patients aged 5 to 90 years.

#### Study characteristics

Table 1 summarizes the characteristics of the included studies [7–9, 11, 12, 33–46]. The review included eighteen quantitative [7, 9, 12, 33–35, 37–41, 43, 47–50] and four qualitative studies [8, 44, 46, 51]. The majority of the studies were from Ethiopia [34, 35, 41, 47, 52], Uganda [8, 12, 46, 49, 51] and India [7, 37, 50]. The rest of the studies were from New Zealand [11, 44], Fiji [39, 40], Pakistan [33, 48], Turkey [36], Egypt [38] and Sudan [9]. Most of the included quantitative studies defined optimal adherence as receiving ≥ 80% of BPG injection doses. BPG prophylaxis adherence ranges from 7 [40] to 92.2% [37].

**Table 1:** Characteristics of the included studies

Authors (location)	Participants and setting	Design and sample	Outcomes measured	Summary of findings	Quality 100%
Adal. 2022 [41] (Ethiopia)	→ RHD patients → Four public hospitals in AA: Tirunesh Beijing Hospital, Zewditu Memorial Hospital, Yekatit 12 Hospital, and Saint Peter Specialized Hospital	→ Quantitative: cross-sectional *→n* = 400	Adherence level: ≥ 80% and associated factors	→The median age was 26.5 years → The majority were females, 59.8% and most were from urban areas (80.1%) → Majority (70.9%) of the participants’ BPG prophylaxis adherence was adequate Facilitators → Adherence to BPG prophylaxis was enhanced by both awareness that bacteria are responsible for RHD and BPG prevents its recurrence and worsening Barriers → Living in rural areas was linked with inadequate adherence → RHD patients residing in a family of more than 5 members and traveling from more than 30 km were inadequately adherent to BPG prophylaxis → Other barriers to BPG adherence were lack of BPG in a follow-up hospital, fear of catching COVID-19, fear of injection pain, and poor awareness of prophylaxis Age, educational status, health insurance, hospital admission history, and comorbidities didn’t affect BPG adherence	100
Adem, 2020 [53](Ethiopia)	→ RHD patients → All age groups, on benzathine penicillin prophylaxis for at least 1 year from four public hospitals in the Jimma zone	→ Quantitative: cross-sectional *→n* = 278 using consecutive sampling	Adherence to BPG: ≥ 80% and associated factors	→ Majority of the participants were females with an average age of 24 years → Good adherence was reported in two-thirds of the participants (63%) Barriers → Adherence to BPG prophylaxis was affected by living in a rural area, coming from a long distance from a health facility(> 30 km), duration of BPG injection (> 5 years), lack of money, and having a mild symptom Sex, age, family size, duration of RHD illness, and hospitalization history were not related to BPG adherence	→ 87.5
Alkan, 2022 [36] (Turkey)	→ Children diagnosed with rheumatic heart disease in Celal Bayar University Faculty of Medicine, Department of Pediatric Cardiology	→ Quantitative: cross-sectional → *n*= 43	Adherence to BPG was considered when administration was missed less 2–3 times per year	→ 43 participants with a mean age was 16.2 ± 2.83 years participated in the study → Adequate adherence was reported in the majority of RHD patients (72%) Barriers → Fear of syringes/injection → Forgetting to get a prescription and/or take the drug when the time comes (*p* < 0.05) Age, gender, living in rural areas, whether the patients received enough information, fear of developing adverse effects, lack of health insurance, or difficulties in reaching hospital not related to the level of adherence	→ 50
Anderson, 2019( [44](New Zealand)	Age: under 40 years RHD pts Self-identified as Māori and/or Pacific hospitalized with a recurrence of acute RF and/or identified with chronic RHD in the North Island District Health Board	→ Qualitative → Semi-structured family/individual interviews with patients and their families → Thematical analysis	Explored barriers to secondary prophylaxis	38 participants interviews Facilitators: → Flexible, community-based models of care → Good communication and rapport with HPs → Good information sharing and referral pathways Barriers: → Models of service delivery, employment commitments, childcare, social obligations, transport access, financial pressure, lack of cultural safety, poor handling of patients, and not friendly/age-specific care	100
Arvind, 2021 [37] India	→ RHD patients → aged 22 years or younger and their families from all India Institute of Medical Sciences (AIIMS), tertiary center in New Delhi	→ Quantitative: cross-sectional *n* = 420 pts and families	Self-reported BPG adherence. BPG Adherence level: ≥ 80% and associated factors	→ The mean age was 11.6 ± 2.9 years → The majority were male, from rural areas, and belonged to lower socioeconomic strata → Most of the participants (92.2%) were adherent Barriers → Many of the participants took BPG from the private clinic and had to walk far → Lack of knowledge regarding the importance of secondary prophylaxis → Misinformation by the treating physician No difference in rural/urban areas, expenses, and distance traveled regarding patient compliance	→ 50
Awan,2021 [48] Pakistan	→ Outpatient and admitted patients of either gender aged 5 to 55 years → National Institute of Cardiovascular Diseases, Karachi, Largest tertiary hospital	→ Quantitative: cross-sectional → *n* = 195	Nonadherence was considered when none of the BPG injections were taken:	→ 195 patients, most were female, 66.7% → The mean age was 32.25 ± .78 years Barriers: → Painful injections (most common reason) → Experience allergic reaction → Lack of access to healthcare in nearby → Injection unavailability at nearby health facility → Friends/family advised otherwise → Felt sick and unable to take injections → Financial constraints	→ 75
Balbaa, 2015 [54] Egypt	→ School children diagnosed with RHD through an Echocardiographic screening program Age: 5–15 years → Aswan Heart clinic	→ Quantitative: cross-sectional *→ n* = 29	Adherence: received over twelve injections (> 75%)	→ RHD patients and or parents/guardians were included in the study and over two-thirds (65.5%) of the participants were adherent Facilitators → Better understanding/knowledge of the disease (RHD) enhanced adherence to BPG prophylaxis → awareness of the effect of missing prophylaxis was related to better adherence to BPG prophylaxis → family appointment reminders Barriers → Most non-adherent RHD patients were intentionally avoiding their BPG injection compared to the adherent patients → Prolonged wait clinic time was the most reported barrier for inadequate BPG prophylaxis adherence	→ 50
Culliford-Semmens 2017 [43] (New Zealand)	→ RHD patients diagnosed by echocardiographic screening in local Counties Manukau, Tairawhiti, Bay of Plenty, Northland, and Capital Coast District Health Boards	*→ n* = 57 RHD patients who were identified through school-based echocardiographic screening	BPG Adherence level good adherence: > 80%	→ Over half of the respondents were males → The majority were males, 53% → The median age was 12 years → Most of the participants were adherent to BPG prophylaxis in the study area Facilitators → RHD registry-based-BPG delivery related to better BPG adherence compared to primary healthcare penicillin delivery	→ 54.54
Edwards 2021 [9] (Sudan)	→ Enrolled patients in a national RHD registry → Aged 12 to 90 years → Al-Shaab Teaching Hospital, and Ahmed Gasim Cardiac and Renal Transplant Teaching Hospital, in Khartoum	→ Quantitative: survey *→n*: 397 → consecutive sampling	BPG adherence and associated barriers and facilitators	→ 397 RHD patients, over three-fourths of the patients were females with a median age were 40 years → Only 32% had good adherence to BPG prophylaxis Facilitators → Younger age, shorter wait time, perceived adequacy of healthcare facility staffing, and treatment costs were considered factors related to enhanced adherence → Perception of improved symptoms with treatment [BPG prophylaxis injection] linked to better adherence → Better household monthly income, educational level, and residence mildly influenced adherence	→ 100
Engelman, 2016 [40] (Fiji)	All RHD cases diagnosed through echocardiographic screening from national:76 health clinics	→ Quantitative: retrospective cohort *→ n* = 494	Adherence was measured using the proportion of days covered (PDC ≥ 0.80)	→ 494 patients were included in the study → Overhalf of the respondents were female (54%) and the median age was 14 years → Adherence: very low level of the respondents had adequate adherence (7%) Facilitators → Living in an urban area is related to better adherence to BPG prophylaxis Barriers → Increasing age and time since diagnosis were related to inadequate adherence to BPG prophylaxis	→ 81.81
Engelman, 2017 [55] (Fiji)	All young diagnosed with RHD through echocardiographic screening in the Central Division of Fiji	→ Quantitative: cross-sectional *→ n* = 101	Adherence level using a structured interview	→ 101 RHD patients with a median age of 17.2 years → A good level of adherence was reported in two-thirds of the study participants (60%) Facilitators → Better adherence was observed in RHD patients who used reminder strategies, particularly phone-based reminders Barriers → Inadequate adherence was related to the distance traveled to receive the injection. Their main reasons are unavailability and logistic reasons of proximity of the clinic to family, school, or employment → Lack of understanding that injections were needed was also linked to inadequate adherence → Other factors reported were feeling well and healthy, transport unavailability, lack of drug supply at the clinic, and pain during injection	→ 87.5
Huck, 2015 [56] (Uganda)	A total of 38 adults (> 26 years) and adolescents/young adults (14 to 26 years) who were part of the RHD registry at the Uganda Heart Institute were included population Participants consented and enrolled using quota sampling methodology from Kampala hospital	Qualitative: focused group discussion. Participants at all levels of adherence were purposively enrolled in 5 groups. Transcripts were analyzed using qualitative description analysis and health behavior models	Barriers and facilitators of adherence to BPG	→ 36 participants in 5 FGD → Most of the participants were females (64%), from an urban area (81%), and had low family income (69%) → Ages ranged from 14 to 58 years → 58% were adherent (> 80% injections) Facilitators: → Worsening of symptoms associated with missing injections, and improvement in how they felt with receiving the injection. Some said that they felt their “heart beating very fast,” “breathing hard,” increased pain in the chest and joints, and fever after missing an injection → Patients also expressed a personal motivation or responsibility to be healthy. A 56-year-old woman said, “You are fully responsible for your life and health” and a 26-year-old woman said, “When you follow the doctor’s recommendation, it helps you prolong your life as well as meet your future ambitions.” → A reminder including a phone reminder, appointment receipts, or medical documents to track injections → Reminders from family or friends → Family provided transportation and medication money and came with participants to their appointments → Participants valued the relationship with healthcare providers and compassion → Proximity to a clinic or living close to a clinic → Many participants continued to get penicillin injections despite their limited understanding of the disease process Barriers: → Lack of resources for medication and transportation → Injections: “extremely painful” → Participants mentioned that family and friends were “tired of treating us,” or thought that those with RHD are “lazy,” or that “heart disease was a death sentence.” → Lack of support material support from family and friends such as transportation or money for appointments and injections → Bad rapport or misunderstandings with health care providers were also mentioned as barriers. For instance, lack of health care provider continuity impaired the patient-provider relationship → Patients expressed that healthcare providers did not always communicate the reason why they needed to take the injections → Poor availability in their local community of skilled health care providers → Providers were not knowledgeable or skilled. For instance, the “doctor/nurse feared to administer [the injection],” assumed they had another disease such as syphilis, “did not mix the drugs with lidocaine,” or told the patient to go to the capital hospital for their injection *→ Participants were also dissuaded by long wait times for appointments.* Participants said, “[you] sit for very long hours” and “you spend a day without seeing the doctor.” → Shortages of penicillin Inaccurate knowledge. A frequently mentioned concept of causation was “overthinking,” “worrying so much,” and “a lot of thinking.”	→ 80
Mehta, 2016 (50] (India)	Patients less than 18 years ago with confirmed RHD and enrolled in Cardiology clinics follow-up at the All-India Institute of Medical Sciences (AIIMS), New Delhi	→ Quantitative: prospective study → *n* = 451	Adherence to BPG prophylaxis adherence: > = 80%	→ Most were male, 66.1% → The median age was 12.2 years → BPG adherence, mean: 93.6% Barriers: perceptions → lack of awareness about SP → fear of injections → injectable BPG unavailable near home → BPG stopped by a local physician after valvular intervention	→ 72.72
Mekonen, 2020 [34] (Ethiopia)	All patients with RHD and receiving BPG prophylaxis at least for 6 months were included in the study Tikur Anbessa Specialized Hospital	→ Quantitative: cross-sectional *→ n* = 145 using convenient sampling	Adherence to BPG: ≥ 80%	→ The study included 145 RHD patients and over three-fourth of them were females with a mean age was 30.12 ± 9.62 years → Most of the participants had in better BPG adherence level, 80.6% Facilitators → Adequate level of adherence to BPG prophylaxis was linked with a history of no hospitalization or one time hospitalization Barriers → Half of RHD patients didn’t know why they took BPG which was also linked to inadequate adherence to prophylaxis → Skipping BPG injection after a missed BPG dose until the next appointment Education, religion, duration of BPG prophylaxis, wait time, knowledge of BPG injection, and reasons for missed doses were not related to BPG adherence	→ 100
Muhammed, 2020 [47] (Ethiopia)	RHD patients enrolled for at least 1-year follow-up at cardiac clinic on BPG prophylaxis at Jimma Medical Center	→ Quantitative: cross-sectional *→ n* = 241	Adherence: 80% or more	→ 241 participants with age ranges between 5 to 68 years were included → Majority were from a rural part → Only 55.2% RHD patients had good adherence Barriers → The inadequate adherence in this study area was due to lack of money, distance from the hospital, fear of medication side effects, painful injection, and lack of knowledge about the disease and prevention	→ 75
Musoke, 2013 [57] (Uganda)	New and old RHD pts Age: 5 to 55 years on prophylaxis for a period of 1 year in Mulago Hospital cardiac clinics	→ Quantitative: Longitudinal study → n = 95 consecutive patients	BPG adherence by self-report Adherence: 80% or more	→ The median age was 28 years. Majority of the participants were females → Residence: near to two-thirds live in urban and the rest live in rural areas which are related to a lower level of BPG adherence → Poor BPG adherence level reported in the study area (54%: ≥ 80% doses) Barriers: → Painful benzathine penicillin injection was reported as the main reason for missing injection followed by lack of transport money → No BPG adherence difference between the RHD passport and the Self-report → No BPG adherence difference between the RHD passport and the Self-report Age, sex, rural/urban residence, educational status, and clinical symptoms were not related to BPG adherence	→ 81.81
Nalubwama, 2023 [46] Uganda	36 sampled RHD pts: contained a balance of gender and age groups from Uganda’s national RHD research registry. Children excluded	Qualitative In-depth interviews were purposively sampled Inductive and deductive methods, with the latter informed by the socioecological model. Thematic content analysis was used	Barriers and facilitators for BPG prophylaxis (RHD care)	Facilitators: → A positive attitude toward life and the desire to care for their families → Social support, including support from partners, families, and communities → inancial support from families/communities → Encouragement and reminders → Good patient care practices by healthcare providers → Participants believed that they were more likely to access care and remain adherent to treatments when they were treated well by their providers → Communicated well and provided information and counseling Barriers: → Injections are very painful → Cost of travel is a major barrier to care. Most participants had to travel long distances for routine care, incurring substantial out-of-pocket costs regularly → Felt better → Poor moral and social support → Weak supply chains and stockouts → Frustration and disappointment at having to travel long distances to health facilities, only to find medication and health workers → Lengthy waiting time → Missing a full day of work to attend their clinics, incurring other expenses like meals → Feeling information and counseling about RHD were inadequate	→ 80
Nemani, 2018 [7] (India)	RHD patients diagnosed at least 1 year ago at Nizam’s Institute of Medical Sciences, Hyderabad	→ Quantitative *→ n* = 500 RHD cohort	BPG adherence: 80% or more	→ Over half (52.2%) of the participants were compliant with BPG prophylaxis Facilitator → History of RF recurrence was reported as an enhancer of adherence to BPG prophylaxis Barriers → Noncompliance to BPG prophylaxis was more common among males, low socioeconomic status, and uneducated RHD patients → Insufficient counseling by 61% and told but neglected (like feeling better, pain, financial and transport problems, shortage of medicines, and allergy) → The most common reason cited for noncompliance was the absence of proper counseling followed by a sense of well-being, injection site pain, and financial constraints However, age, rural residence religion, acute rheumatic fever at presentation, and severity of RHD didn’t have a significant relation with adherence rate	→ 100
Okello, 2017 [49] (Uganda)	Participants aged 5–60 years with established RHD in Mulago National Referral Hospital in Kampala	→ Quantitative: a prospective cohort study *→ N* = 449	BPG prophylaxis adherence: ≥ 80%	→ most were female, 66.8% → The median age was 30 years → BPG adherence: 57.5% Barriers: → Increasing age (71% of those of those < 15 years compared to those > 50 years → No formal education → Presence of comorbidities such as the presence of stroke, atrial fibrillation	→ 54.54
Sial, 2018 [33] Pakistan	RHD patients diagnosed at least 1 year ago and seen at OPD or inpatient at the Cardiology Department of Chandka Medical College, Larkana	RHD patients. The sample size was not calculated	BPG adherence: 80% or more	→ Close to 70% of the participants were females, and their mean age was 34.09 years → Almost three-fourths of the patients were adherent to BPG prophylaxis Barriers → patients presenting to the emergency department had a lower adherence rate → Age over 30 years Adherence level was also related to the type of heart valve lesion → Age over 30 years is linked to inadequate adherence → Presence of heart valve lesions was associated with an inadequate level of adherence to BPG prophylaxis	→ 50
Voleti, [58] 2020 Uganda	Mothers with RHD at a median postpartum time of 2.5 years after delivery in Central and Eastern Uganda	→ Qualitative *→ n* = 40	BPG adherence: ≥ 80%	→ Adherence to BPG prophylaxis was poor in 70% Barriers → Financial limitations → Transportation cost/availability → Fear of injection → Cost of medication → Lack of healthcare workers → Distance to higher-level facilities → Misunderstanding: health literacy: “I used to sell charcoal, we could go and buy it from far places, climb those Lorries, and I think that is where I got this disease from.”	→ 100
Zewde, 2022 [52] Ethiopia	Adults RHD patients on benzathine penicillin prophylaxis in Tikur Anbessa Specialized Hospital	→ Quantitative: Cross-sectional *→ n* = 385	BPG prophylaxis Adherence: 80% or more	→ 385 RHD patients were included mostly females, 71.7% with a mean age was 31 years → Majority of the RHD patients had adequate BPG adherence, 77.9% Facilitators → Majority of the respondents were knowledgeable about RHD’s that could facilitate better adherence to BPG prophylaxis → Over three-fourths, believed that BPG prevents recurrence → One-third believed that BPG cures RHD → Close to one-tenth, believed that BPG prevents RHD progression Barriers → BPG unavailability, older age, missing appointments, and BPG injection refusal by a healthcare provider, forgetting the BPG schedule were linked with poor adherence Fear of injection was not related to any level of adherence	→ 100

## Main findings

The identified barriers and facilitators of BPG prophylaxis adherence are summarized and mapped onto the six COM-B components in Table 2. The COM-B model and its components were used to determine the barriers to and facilitators of BPG prophylaxis adherence. Our review identified 53 factors (33 barriers and 20 facilitators) associated with BPG adherence from the 22 included studies. All of the findings were mapped onto all six COM-B model components.

**Table 2:** Overview of results: summary of barriers and facilitators across domains

COM-B constructs	Barriers [Barrier code]	Source	Facilitators [Facilitator code]	Source
Physical capability	Older age [B1]	[9, 40, 49, 52]	Female[F1]	[7]
	Felt sick and couldn’t come for BPG injection [B2]	[30, 46]	Previous symptomatic RF [F2]	[7, 34]
	Comorbidities [B3]	[49]		
	Emergency room visit [B4]	[33]		
	Felt health and well[B5]	[12, 39, 52, 57]	Severe RHD[F3]	[33]
	Longer duration of RHD diagnosis [B6]	[40, 53, 59]		
	Long duration of BPG prophylaxis[B7]	[53]		
	Mild RHD severity [B8]	[34, 59]		
Psychological capability	Lack of education or knowledge/awareness understanding on BPG prophylaxis[B9]	[7, 9, 37, 39, 41, 49]	RHD knowledge or better understanding of the disease [F4]	[38]
	Misconception or poor RHD knowledge [B10]	[8, 9, 46, 47, 58]		
	Forgetfulness [B11]	[34, 36, 52]		
Physical opportunity	Transport cost [B12]	(7,8,12,46,52,60]	Adequate healthcare coverage and perceived adequacy of healthcare staffing [F5]	[9, 44]
	Transport unavailability [B13]	[9, 39, 44, 52, 58]		
	prolonged clinic waiting time[B14]	[8, 9, 38, 44, 52]		
	Employed/busy[B15]	[46]	RHD registry [F6]	[42–44]
	Unavailability of BPG[B16]	[7, 8, 37, 39, 41, 48, 50, 52]	Urban residence [F7]	[39, 41, 53]
	Cost of medication/treatment [B17]	[8, 9, 44, 51]	Reminders: system-based (health clinic cards, phone calls) [F8]	[8, 39, 45, 46]
	Unavailability healthcare providers[B18]	[8, 37, 48, 51]	Community based care[F9]	[44]
	Distance from health care[B19]	[8, 39, 41, 46, 47, 51, 53]	School and home-based BPG delivery [F10]	[44]
	Rural residence[B20]	[41, 53]		
	Financial pressure/constraints [B21]	[7, 44, 47, 48, 51]		
	Residing in a family of 5 or more [B22]	[41]		
Social opportunity	Poor relationship with family, friends, and healthcare providers[B23]	[8, 9]	Family reminder[F11]	[8, 38, 45]
	Misunderstanding of provided information [B24]	[8, 37, 44]	Support from family/friend [F12]	[8, 9]
	Inadequate counseling and information about RHD/BPG[B25]	[7, 9, 46]	positive interaction between patient healthcare providers[F13]	[8, 44]
	Family/friend advice [B26]	[48]	Positive influence from other success [of treatment] [F14]	[9]
Reflective motivation	Perception of knowledge or skill gap healthcare/incompetence [B27]	[8, 9]	Worsening symptoms with missing injections, and improvement in how they felt with receiving the injection[F15]	[8]
	Poor patient handling during care and BPG injection[B28]	[34, 44]	Awareness of the consequence of missing BPG prophylaxis[F16]	[8, 38]
	Healthcare providers refuse to provide BPG injections [B29]	[37, 52]	Absence hospital admission[F17]	[7, 34]
	Intentional avoidance of BPG injection[B30]	[38]	Perceived improved symptoms with BPG [F18]	[8, 9]
	Perception of better care in referral or higher health facility[B31]	[9, 37]	Personal motivation to self-support or family support [F19]	[8, 9]
Automatic motivation	Fear/painful BPG injection[B32]	[8, 9, 12, 36, 37, 39, 41, 46–48, 50–52]	Reduction of BPG injection pain by mixing it with analgesics [F20]	[52]
	Experience of allergic reaction/side effects [33]	[47, 48]		

## Barriers to BPG adherence

### Physical capability

In this review, older individuals, a longer duration of prophylaxis, and feeling healthy were commonly identified as physical capability barriers across the studies. Five studies included in this review revealed that older RHD patients had inadequate adherence to BPG prophylaxis [9, 40, 42, 49, 52]. For example, in one of the studies, those over 50 years of age had lower BPG adherence compared to the younger patients [49]. Participants who felt healthy had suboptimal levels of BPG prophylaxis adherence. They did not properly attend their regular BPG prophylaxis [12, 39, 52]. Others who felt sick on the day of clinic visits or appointments did not attend appointments to receive their BPG injection due to feeling too sick to travel [12, 33]. The longer the duration of RHD diagnosis was the barrier to BPG adherence [40, 53]. Other identified physical capability barriers were the presence of comorbidities such as stroke or arterial fibrillation [49] and emergency admission to the hospital [33].

### Psychological capability

Low awareness or inadequate knowledge of RHD/BPG prophylaxis was linked to inadequate BPG prophylaxis adherence [7–9, 37, 39, 41, 46, 49, 56]. For instance, in a study conducted in Uganda, “overthinking,” “worrying so much,” and “a lot of thinking” were mentioned as the causes of RHD [8]. Many participants continued to receive penicillin injections despite their limited understanding of the disease process [8]. Half of the participants from one study did not know why they were receiving BPG prophylaxis [34]. In addition, in five of the included studies, participants’ misconception/misunderstanding was related to inadequate BPG adherence [9, 46, 52, 56, 58]. In another study, participants skipped their BPG injection after a missed BPG dose until the next appointment [52]. Participants from two studies also reported that forgetfulness of receiving a prescription, taking the drug, or schedules were the main reasons for inadequate BPG adherence [36, 52].

### Physical opportunity

Among the 22 studies included in this review, transportation-related barriers were discussed in 10. The cost of transportation [7, 8, 12, 46, 49, 52, 58] and the unavailability of transportation [9, 39, 44, 52, 58] remained major barriers, which were overlapping and dual burdens [52, 58]. A prolonged clinic wait time during regular BPG injection visits was a commonly discussed barrier [8, 9, 44, 46, 52, 54]. Records included in this review identified a shortage or unavailability of BPG as the main reason for RHD patients’ inadequate BPG adherence [7, 8, 37, 39, 41, 48, 50, 52]. On the other hand, the cost of medication is a barrier to accessing regular BPG injections [8, 9, 44, 58]. Financial problems or constraints created challenges in accessing BPG injections [7, 44, 49, 50, 58]. Distance from healthcare was a commonly discussed barrier [8, 39, 41, 46, 47, 53, 58]. Participants also expressed their frustration and disappointment about traveling long distances to receive both healthcare providers’ care and BPG injections [46, 58]. In addition, the unavailability of skilled healthcare providers in local communities was mentioned; therefore, RHD patients had to travel far to receive monthly BPG injections [8, 37, 46]. Living in rural areas was also associated with inadequate BPG adherence [41, 53].

### Social opportunity

The included studies identified different forms of communication barriers that made RHD patients less engaged in BPG prophylaxis [8, 37, 44, 48]. RHD patients were misinformed or misunderstood about the information provided by their healthcare providers and/or friends to stop receiving their BPG injection [8, 37, 44]. Participants have also commented on the presence of misunderstanding or inadequate communication [8]. Another study discussed advice from family/friends to stop BPG injection as a barrier [48]. Poor relationships between healthcare providers and RHD patients affected engagement in BPG prophylaxis injection [8, 9]. Inadequate counseling from healthcare providers was documented as a barrier [7, 9]. Another study reported that the information and counseling services provided by healthcare providers about RHD were inadequate as perceived by patients [46].

### Reflective motivation

A lack of trust in healthcare providers’ skills was identified and discussed as a barrier to BPG prophylaxis adherence [8, 9]. Participants commented that healthcare providers were not knowledgeable or skilled. They [healthcare providers] “feared to administer [the injection]” or told the patient to go to other hospitals for injection [8]. The intention to look for better healthcare (such as referral hospitals) as a result of avoidance of follow-up in general healthcare facilities was discussed as a barrier [9, 37]. Participants discussed improper patient handling, such as a lack of cultural safety and a lack of friendly healthcare providers, as a barrier [34, 44]. Healthcare providers’ refusal of BPG injection was reported in studies included in this review [37, 52]. On the other hand, intentional refusal of regular BPG injections by RHD patients was reported [38].

### Automatic motivation

In more than 50% of the studies included, most of the participants avoided BPG injection due to fear of pain or painful BPG injection [8, 9, 12, 36, 37, 39, 41, 46, 47, 49, 52, 58]. In two other studies, fear of allergic reactions and/or side effects mentioned motivational barriers [47, 48].

## Facilitators of BPG adherence

### Physical capability

The severity of RHD [33] and previous symptoms of rheumatic fever [7] seem to have reinforced participants’ ability to receive regular BPG prophylaxis injections. Participants from the included study expressed a personal motivation or responsibility to be physically healthy. A 56-year-old woman said, “You are fully responsible for your health”, and a 26-year-old woman said, “When you follow the doctor’s recommendation, it helps you prolong your life as well as meet your future ambitions” [8]. Female participants were better at maintaining an optimal level of adherence to BPG prophylaxis [7]. In addition, younger age was associated with better adherence [9].

### Physical opportunity

Access to healthcare and perception of healthcare provider adequacy by participants were discussed as facilitators of BPG prophylaxis adherence [9, 44]. System-based data management and RHD patient follow-up facilitated BPG prophylaxis adherence. Enrollment in the RHD registry either encouraged participants or eased monitoring and feedback [43, 44]. Participants from urban areas were better able to receive regular BPG injections than were rural residents [39, 41, 53]. Different reminder forms were discussed, and many participants commented that the presence of reminders enabled them to regularly attend their monthly BPG injections. For instance, reminders from health care systems, such as clinic cards and phone calls, were appreciated as facilitators in four studies [8, 38, 39, 46]. School and home-based BPG delivery were discussed as facilitators of BPG prophylaxis adherence [44].

### Social opportunity

Reminders received from family/friends enabled RHD patients to receive regular BPG injections [8, 38]. Support from family, friends, or community members enabled RHD patients to receive BPG injections [8, 9]. A positive relationship between RHD patients and their healthcare providers facilitated BPG injection uptake [8]. Moreover, the treatment success of peers encouraged RHD patients to adhere [9].

### Reflective motivation

Awareness of the consequence of missing medication as a motivator of BPG prophylaxis injection [8, 38]. In other studies, the absence of hospital admission motivated RHD patients to adhere better to regular BPG injections. At the same time, these participants were motivated by a previous history of symptomatic rheumatic recurrence [7, 34]. It seems that RHD patients with a history of illness are motivated by their unpleasant experience of rheumatic recurrence. Participants from other studies were also motivated by their intention to support their family or themselves [8, 9]. Perceived improvement in symptoms following BPG injection has also enhanced adherence to BPG prophylaxis [8, 9].

### Automatic motivation

A study indicated that the reduction in BPG injection pain caused by mixing with analgesics such as lidocaine motivated participants to receive regular BPG injections [52]. In another study, participants frequently associated the worsening of symptoms of acute rheumatic fever with missing a single BPG injection and improvement in how they felt as soon as they received the injection [8].

## Discussion

This review aimed to identify the barriers to and facilitators of BPG adherence among RHD patients. This review identified different barriers and facilitators using an established behavioral model [the COM-B]. The utilization of the COM-B model to guide systematic reviews on the barriers to and facilitators of BPG adherence is the first of its kind. Nearly all factors identified in this study were fitted into the six categories of the COM-B model. The COM-B model with its behavioral change wheel (BCW) was used to discuss our findings in light of the intervention functions [16].

Capability barriers such as lack of awareness or inadequate knowledge of RHD and BPG prophylaxis and misconception/misunderstanding remain crucial and are linked to various barriers. For instance, those who felt healthy remained less adherent, which could be related to inadequate knowledge and could be addressed through the education and training functions of BCWs. Patients with better knowledge or awareness of BPG prophylaxis or RHD conditions were more adherent to BPG prophylaxis [38]. Hence, attention should be given to education and training to further enable RHD patients to maintain an optimal level of adherence. Forgetfulness also remains a critical challenge in BPG prophylaxis adherence [10, 42], which is consistent with our review findings. Interventions that target forgetfulness could also benefit older patients and those with longer prophylaxis durations. These can include designing remainders or recall systems [phone calls, text, RHD hotlines] from the healthcare system or social environment is a recommended enablement function to improve BPG adherence. Being male was associated with lower BPG adherence. This can be addressed by the education and persuasion function of BCWs. Male patients should be informed of the consequences of poor BPG adherence.

Opportunity barriers such as the unavailability and/or cost of BPG medication, long clinic wait times, transportation, distance, healthcare provider miscommunication, and inadequate counseling can be addressed through physical and social environmental changes [16, 24]. Although BPG remains an essential drug, its unavailability and cost remain major barriers across the studies included in our review. Adding objects to the environment [in this case, BPG supply] is an essential aspect of restructuring the physical environment in the BCW intervention function to ensure drug availability. Therefore, an adequate BPG supply and a waiving cost of BPG should be ensured in environments with high rates of rheumatic recurrence or RHD. To reduce the clinic waiting time and long-distance travel, which are common barriers across studies, the healthcare system should restructure the physical environment, which includes decentralization of BPG injections to community-level healthcare providers. Our recommendation is in support of previous studies [9, 13, 14]. Persuasion functions such as providing information about health consequences or feedback on behaviors should be an integral part of the BPG prophylaxis program to clear out miscommunication about RHD/BPG. The education and persuasion intervention functions of BCWs should be used by healthcare providers to address inadequate counseling. Healthcare providers should give due emphasis to the consequences of poor adherence to BPG during each patient visit. Our recommendation is consistent with the medication adherence recommendation [15, 60].

Motivation is a key behavioral factor in BCW, and it improves one’s ability to maintain desired behavior [in our case, BPG adherence] [24]. In our review, fearful/painful BPG injection remained the most common motivational barrier. This factor can be addressed by a persuasion, education, and enablement intervention function of the BCW. Thus, healthcare providers should provide information on the health consequences of missing BPG injections in a way that RHD patients clearly understand the benefits of BPG injections. To reduce negative emotions and enhance experience, it may be essential to use lidocaine to reduce pain during BPG injections. Lidocaine was found to be effective at reducing pain related to BPG injections in randomized controlled trials [61, 62]. A lack of trust in healthcare and a perception of poor handling were also identified as demotivators and can be addressed by the persuasion and enablement intervention functions of BCWs. Healthcare providers should demonstrate effective therapeutic communication and build positive relationships with RHD patients. Our recommendation is in line with a systematic review of communication strategies to improve medication adherence [15].

### Strengths and limitations

Our review included both quantitative and qualitative studies to better understand the factors affecting BPG prophylaxis adherence. To the best of our knowledge, this is the first systematic review that evaluated the barriers to and facilitators of BPG adherence among RHD patients using the COM-B theoretical framework, pointing to possible intervention functions. Despite these strengths, this study has several limitations. The identified barriers and facilitators were identified from RHD patients’ perspectives. The views of healthcare providers were not included. The inclusion of studies limited to the English language could not capture factors addressed in other languages. Although our search strategy was broad and comprehensive, it was limited to only peer-reviewed publications.

### Recommendations for practice

The findings from this review can be used to design an implementation strategy to improve BPG prophylaxis adherence. The review identified different barriers across the three domains of the COM-B model which could addressed by using BCW intervention functions and behavioral change techniques. Hence, the lack of information about BPG prophylaxis could be addressed by the provision of adequate information about BPG prophylaxis by healthcare providers. Whenever possible, patients’ concerns should be addressed through education and effective communication. Healthcare providers should establish a positive communication environment. A positive communication environment could also enhance a trust relationship between patients and BPG prophylaxis providers which is a key step in improving uptake of the prophylaxis. Adequate and regular education and counseling should be given to clarify misunderstandings/misconceptions about patients’ conditions. Whenever possible, BPG injections should be decentralized to community healthcare settings to reduce the cost of travel, clinic wait time, and related out-of-pocket expenses. Lidocaine may also be considered to reduce pain during BPG injection. Healthcare reminder systems [such as phone calls, text, and RHD hotlines] or social reminder systems [family or friend reminders] should be ensured. Patients with RHD are expected to mobilize available community resources and become motivated to receive BPG injections and improve their well-being. Finally, addressing capability, opportunity and motivational barriers should be a continued and essential process.

## Conclusions

Our review revealed variable levels of BPG adherence across studies and revealed significant facilitators of and barriers to prophylaxis adherence. We used the behavioral change theoretical framework to synthesize findings around barriers and facilitators. The COM-B model with BCW helped us craft theory-informed interventions to improve BPG prophylaxis adherence among RHD patients. Besides, the Taxonomy of Behavioural Change Techniques helped us to describe the content and approaches of intervention to address the identified barriers. Further research is recommended to identify contextual interventions to address barriers and capitalize on facilitators.

## Supplementary Information

Additional file 1: Sample search.

Additional file 2: Critical appraisal scores.

Additional file 3: Table S3. Credibility of included studies.

Additional file 4: Table S4: Data transformation.

## Data Availability

The datasets used during the current review are available from the corresponding author upon reasonable request.
