# Supplementary material for: Barriers and facilitators of benzathine penicillin G adherence among rheumatic heart disease patients: a mixed methods systematic review using the COM-B (capability, opportunity, and motivation for behavior) model
Source: Syst Rev. 2024 Dec 3;13:297. doi: 10.1186/s13643-024-02691-1 (PMC11613468; doi:10.1186/s13643-024-02691-1)
Supplement: Supplementary file 1 — Additional file 1: Sample search. [file 13643_2024_2691_MOESM1_ESM.docx]

**Supplement 1: Sample search**

**Search strategy.**

| **Population** | **Intervention** | **Outcomes** |
| --- | --- | --- |
| “rheumatic fever” OR “rheumatic heart disease” | “Secondary prophylaxis” OR “secondary prevention” OR “benzathine penicillin G” OR “penicillin G benzathine” OR “benzathine penicillin” OR “benzathine benzylpenicillin” OR “disease management” OR “management” | “Patient compliance” OR “compliance” OR “noncompliance” OR “noncompliance” OR “treatment refusal” OR “guideline adherence” OR “medication adherence” OR “adherence” OR “nonadherence” OR “nonadherence” OR “alignment” OR “nonalignment” OR “nonalignment”. |

**PubMed**

(“rheumatic fever” OR “rheumatic heart disease”) AND (“secondary prophylaxis” OR “secondary prevention” OR “benzathine penicillin G” OR “penicillin G benzathine” OR “benzathine penicillin” OR “benzathine benzylpenicillin” OR “disease management” OR “management”) AND (“patient compliance” OR “compliance” OR “noncompliance” OR “noncompliance” OR “treatment refusal” OR “guideline adherence” OR “medication adherence” OR “adherence” OR “nonadherence” OR “nonadherence” OR “alignment” OR “nonalignment” OR “nonalignment”).

**Global Health database search**

[All: "rheumatic fever"] OR [All: "rheumatic heart disease"] AND [All: "secondary prophylaxis"] OR [All: "secondary prevention"] OR [All: "benzathine penicillin g"] OR [All: "penicillin g benzathine"] OR [All: "benzathine penicillin"] OR [All: "benzathine benzylpenicillin"] OR [All: "disease management"] OR [All: "management"] AND [[All: "patient compliance"] OR [All: "compliance"] OR [All: "non-compliance"] OR [All: "noncompliance"] OR [All: "treatment refusal"] OR [All: "guideline adherence"] OR [All: "medication adherence"] OR [All: "adherence"] OR [All: "non-adherence"] OR [All: "nonadherence"] OR [All: "alignment"] OR [All: "non-alignment"] OR [All: "nonalignment"] AND [CABI Products: Global Health].
