# Supplementary material for: Barriers and facilitators of benzathine penicillin G adherence among rheumatic heart disease patients: a mixed methods systematic review using the COM-B (capability, opportunity, and motivation for behavior) model
Source: Syst Rev. 2024 Dec 3;13:297. doi: 10.1186/s13643-024-02691-1 (PMC11613468; doi:10.1186/s13643-024-02691-1)
Supplement: Supplementary file 2 — Additional file 2: Critical appraisal scores. [file 13643_2024_2691_MOESM2_ESM.docx]

**Supplement 2: Critical appraisal scores**

**Critical Appraisal of Eligible Analytical Cross-Sectional Studies**

| **Citation** | **Q1** | **Q2** | **Q3** | **Q4** | **Q5** | **Q6** | **Q7** | **Q8** | **Quality score** |
| --- | --- | --- | --- | --- | --- | --- | --- | --- | --- |
| Adem et al. 2020 (35) | N | Y | Y | Y | Y | Y | Y | Y | 87.5% |
| Akan et al. 2022 (36) | N | Y | N | Y | N | N | Y | Y | 50% |
| Arvind et al 2021 (37) | N | Y | Y | Y | N | N | N | Y | 50% |
| Balbaa et al. 2015 (38) | N | Y | Y | N | N | N | Y | Y | 50% |
| Edwards et al, 2021 (9) | Y | Y | Y | Y | Y | Y | Y | Y | 100% |
| Engelman et al 2017 (39) | Y | Y | Y | Y | N | Y | Y | N | 87.5% |
| Adal et al., 2022 (41) | Y | Y | Y | Y | Y | Y | Y | Y | 100% |
| Awan et al 2021(48) | Y | Y | Y | Y | N | N | Y | Y | 75% |
| Zewde et al 2022 (52) | Y | Y | Y | Y | Y | Y | Y | Y | 100% |
| Nemani et al 2018 (7) | Y | Y | Y | Y | Y | Y | Y | Y | 100% |
| Sial et al.2018 (33) | Y | Y | Y | Y | N | N | N | N | 50% |
| Mohammed et al 2020 (47) | Y | Y | Y | Y | N | N | Y | Y | 75% |
| Mekonen et al 2020 (34) | Y | Y | Y | Y | Y | Y | Y | Y | 100% |
| % | 69.23 | 100 | 92.3 | 92.3 | 46.2 | 53.8 | 84.6 | 84.6 |  |

***Q1****: Were the criteria for inclusion in the sample clearly defined? Q2: Were the study subjects and the setting described in detail?*

*Q3: Was the exposure measured in a valid and reliable way? Q4: Were objective, standard criteria used for measurement of the condition? Q5: Were confounding factors identified? Q6: Were strategies to deal with confounding factors stated? Q7: Were the outcomes measured in a valid and reliable way? Q8: Was appropriate statistical analysis used?*

**Critical Appraisal of the Eligible Cohort Study**

| **Citation** | **Q1** | **Q2** | **Q3** | **Q4** | **Q5** | **Q6** | **Q7** | **Q8** | **Q9** | **Q10** | **Q11** | **quality** |
| --- | --- | --- | --- | --- | --- | --- | --- | --- | --- | --- | --- | --- |
| Culliford et al., 2017 (43) | U | N | Y | N | U | N | Y | Y | Y | Y | Y | 54.54% |
| Engelman et al.,2016 (40) | Y | N | Y | Y | U | Y | Y | Y | Y | Y | Y | 81.81% |
| Mehta A, et al 2016 (50) | Y | Y | Y | Y | N | N | Y | Y | Y | Y | Y | 72.72% |
| Musoke et al 2013 (12) | Y | N | Y | Y | N | Y | Y | Y | Y | Y | Y | 81.81% |
| Okello et al 2017 (49) | N | N | Y | Y | N | N | Y | Y | Y | Y | Y | 54.54% |
| % | 60 | 20 | 100 | 80 | 0.0 | 40 | 100 | 100 | 100 | 60 | 100 |  |

***Q1:*** *Were the two groups similar and recruited from the same population? Q2: Were the exposures measured similarly to assign people to both exposed and unexposed groups? Q3: Was the exposure measured in a valid and reliable way? Q4: Were confounding factors identified? Q5: Were strategies to deal with confounding factors stated? Q6: Were the groups/participants free of the outcome at the start of the study (or at the moment of exposure)? Q7: Were the outcomes measured in a valid and reliable way? Q8: Was the follow up time reported and sufficient to be long enough for outcomes to occur? Q9: Was follow up complete, and if not, were the reasons to loss to follow up described and explored? Q10: Were strategies to address incomplete follow up utilized? Q11: Was appropriate statistical analysis used?*

**Critical Appraisal of Eligible Qualitative Research**

| **Citation** | **Q1** | **Q2** | **Q3** | **Q4** | **Q5** | **Q6** | **Q7** | **Q8** | **Q9** | **Q10** | **Total** |
| --- | --- | --- | --- | --- | --- | --- | --- | --- | --- | --- | --- |
| Anderson, et al., 2019 (44) | Y | Y | Y | Y | Y | Y | Y | Y | Y | Y | 100% |
| Huck., et al., 2015 (8) | N | Y | Y | Y | Y | Y | U | Y | Y | Y | 80% |
| Nalubwama, et al., 2023(46) | N | N | Y | Y | Y | Y | Y | Y | Y | Y | 80% |
| Volti, et al., 2020 (51) | Y | Y | Y | Y | Y | Y | Y | Y | Y | Y | 100% |
| % | 50 | 75 | 100 | 100 | 100 | 100 | 75 | 100 | 100 | 100 |  |

**Q1**: Is there congruity between the stated philosophical perspective and the research methodology? Q2: Is there congruity between the research methodology and the research question or objectives? Q3: Is there congruity between the research methodology and the methods used to collect data? Q4: Is there congruity between the research methodology and the representation and analysis of data? Q5: Is there congruity between the research methodology and the interpretation of results? Q6: Is there a statement locating the researcher culturally or theoretically? Q7: Is the influence of the researcher on the research, and vice versa, addressed? Q8: Are participants and their voices adequately represented? Q9: Is the research ethical according to current criteria or, for recent studies, and is there evidence of ethical approval by an appropriate body? Q10: Do the conclusions drawn in the research report flow from the analysis, or interpretation,
