# Supplementary material for: Barriers and facilitators of benzathine penicillin G adherence among rheumatic heart disease patients: a mixed methods systematic review using the COM-B (capability, opportunity, and motivation for behavior) model
Source: Syst Rev. 2024 Dec 3;13:297. doi: 10.1186/s13643-024-02691-1 (PMC11613468; doi:10.1186/s13643-024-02691-1)
Supplement: Supplementary file 3 — Additional file 3: Table S3. Credibility of included studies. [file 13643_2024_2691_MOESM3_ESM.docx]

**Table S3. Credibility of included studies**

| **Article** | Finding | **Illustrations** | **Level of**  **credibility** |
| --- | --- | --- | --- |
|  | **A1** | **Physical capability: barrier of BPG adherence** | ***U*** |
| Musoke et al 2013 (12) |  | Felt health and well |  |
|  |  | Felt sick and couldn't come for BPG injection |  |
|  | **A2** | **Authomatic motivation: barrier of BPG adherence** | ***C*** |
|  |  | Painful benzathine penicillin injection |  |
|  | **A3** | **Physical opportunity: barrier of BPG adherence** | ***C*** |
|  |  | Lack of transport money, away from home |  |
|  | **A4** | **Social opportunity: barrier of BPG adherence** | ***C*** |
|  |  | Advise from friends to stop BPG |  |
| Adem et al. 2020 (35) | **B1** | **Physical opportunity: barrier** | ***U*** |
|  |  | Rural residences, long distances from a health facility. |  |
|  | **B2** | **Physical capability: barrier** | ***C*** |
|  |  | Long duration on BPG injection and mild symptoms |  |
|  | **B3** | **Physical opportunity: barrier** | ***U*** |
|  |  | Household with more than five people |  |
| Culliford et al., 2017 (43) | **C1** | **Physical opportunity facilitator of BPG adherence** | ***U*** |
|  |  | RHD registry based-BPG delivery |  |
| Akan et al. 2022 (36) | **D1** | **Psychological capability: barrier** | ***C*** |
|  |  | Forgetting to get a prescription and/or take the drug when the time comes |  |
|  | **D2** | **Reflective motivation: barrier** | ***C*** |
|  |  | Fear of injection syringes |  |
| Arvind et al 2021 (37) | **E1** | **Automatic motivation: barrier** | ***C*** |
|  |  | Fear of injection pain |  |
|  | **E2** | **Physical opportunity: barrier** | ***C*** |
|  |  | BPG unavailability in the health facility, non-availability of a doctor, non-availability of a doctor |  |
|  | **E3** | **Psychological capability: barrier** | ***U*** |
|  |  | Lack of knowledge on BPG prophylaxis |  |
| Balbaa et al. 2015 (38) | **F1** | **Reflective motivation: barrier** | ***C*** |
|  |  | Intentional avoidance of BPG injection |  |
|  | **F2** | **Pyschologica capability: facilitator** | ***U*** |
|  |  | RHD knowledge |  |
|  | **F3** | **Physical opportunity: barrier** | ***C*** |
|  |  | Prolonged clinic wait time |  |
|  | **F4** | **Reflective motivation: facilitator** | ***C*** |
|  |  | Awareness of the effect of missing BPG prophylaxis |  |
|  | **F5** | **Social opportunity: facilitator** | ***U*** |
|  |  | Family appointment reminders |  |
| Edwards et al, 2021 (9) | **G1** | **Physical opportunity: facilitator** | ***U*** |
|  |  | Shorter clinic wait time, perceived adequacy of healthcare facility staffing, increased treatment costs |  |
|  | **G2** | **Pyschological capability: barrier** | ***C*** |
|  |  | Misconception/poor RHD disease understanding |  |
| Engelman et al 2017 (39) | **H1** | **Physical opportunity barrier** | ***C*** |
|  |  | Transport unavailability and lack of drug supply at the clinic. |  |
|  | **H2** | **Physical capability barrier** | ***C*** |
|  |  | Feeling well and healthy. |  |
|  | **H3** | **Automatic motivation barrier** | ***C*** |
|  |  | BPG injection pain |  |
|  | **H4** | **Social opportunity facilitator** | ***U*** |
|  |  | Reminder strategies, particularly phone-based reminders |  |
|  | **H5** | **Phyical oppotunity barrier** | ***U*** |
|  |  | Their main reasons are unavailability and logistic reasons of proximity of the clinic to family, school, or employment. |  |
|  | **H6** | **Psychological capability barrier** | ***C*** |
|  |  | Lack of understanding on the need of BPG injection |  |
| Adal et al., 2022 (41) | **I1** | **Psychological capability barrier** | ***C*** |
|  |  | Poor awareness of prophylaxis. |  |
|  | **I2** | **Physical opportunity barrier** | ***C*** |
|  |  | Rural residence, residence more than 30 km from a health facility, lack or shortage of BPG in a follow-up hospital |  |
|  | I3 | **Social Opportunity Barrier** | ***C*** |
|  |  | Residing in a family of more than 5 members |  |
|  | I4 | **Automatic Motivation Barrier** | ***C*** |
|  |  | Fear of catching COVID-19, fear of injection pain |  |
| Awan et al 2021(48) | J1 | **Automatic reflection barrier** | ***C*** |
|  |  | Painful injections (most common reason)  experience allergic reaction |  |
|  | J2 | **Physcal opporunity barrier** | ***C*** |
|  |  | Lack of access to healthcare in nearby  Injection unavailability at nearby health facility  financial constraints |  |
|  | J3 | **Physical capability barrier** | ***U*** |
|  |  | Felt sick and unable to take injections. |  |
|  | J4 | **Social opportunity barrier** | ***U*** |
|  |  | Friends/family advised otherwise |  |
| Zewde et al 2022 (52) | K1 | **Reflective motivation facilitator** | ***C*** |
|  |  | Reduction of BPG injection pain by mixing it with analgesics | ***C*** |
|  | K2 | **Physcial opportunity barriers** |  |
|  |  | BPG unavailability, Cost of BPG, unavailability of transport, transport cost, BPG injection refusal by healthcare providers, being busy | ***C*** |
|  | **K3** | **Automatic motivation barrier** | ***C*** |
|  |  | Fear of BPG side effect, BPG injection pain |  |
|  | K4 | Psychological capability barrier | ***C*** |
|  |  | BPG injection forgetfulness |  |
|  | K5 | Physical capability barrier | ***C*** |
|  |  | Older age, felt well and healthy |  |
| Nemani et al 2018 (7) | M1 | **Reflective motivation barrier** | ***C*** |
|  |  | Neglect of the counselling given by the HCP |  |
|  | M2 | **Social opportunity barrier** | ***C*** |
|  |  | Inadequate counselling by HCP |  |
|  | M3 | **Physical capability barrier** | ***C*** |
|  |  | Being male, history of rheumatic fever recurrence |  |
|  | M4 | **Physical opporunity barrier** | ***U*** |
|  |  | Financial and transport problems, and shortage of medicines, low socioeconomic status |  |
|  | M5 | **Psychological capability barrier** | ***C*** |
|  |  | Lack of education |  |
| Sial et al.2018 (33) | N1 | **Physical capability barrier (U)** | ***U*** |
|  |  | Emergency room visit, severe RHD |  |
| Mohammed et al 2020 (47) | O1 | **Physical opportunity barrier** | ***C*** |
|  |  | Lack of money  Distance from hospital. |  |
|  | O2 | **Automatic reflection barrier** | ***C*** |
|  |  | Fear of medication side effects, painful injection |  |
|  | O3 | **Psychological cabability barrier** | ***U*** |
|  |  | Lack of knowledge about the disease and prevention. |  |
| Mekonen et al 2020 (34) | P1 | **Physical capability facilitator** | ***U*** |
|  |  | No or one history of hospital admission |  |
|  | P2 | **Reflective motivation barrier** | ***C*** |
|  |  | Lack of action after missed BPG injection date until next clinic follow up date |  |
| Engelman et al.,2016 (40) | Q1 | **Physical opportunit facilitator** | ***U*** |
|  |  | urban residence |  |
|  | Q2 | Physical capability barrier | ***C*** |
|  |  | increasing age and long time since RHD diagnosis |  |
| Mehta A, et al 2016 (50) | R1 | **Psycological capability barrier** | ***U*** |
|  |  | lack of awareness about SP |  |
|  | R2 | **Automatic refelction barrier** | ***U*** |
|  |  | fear of injections |  |
|  | R3 | **Physical opportnity barrier** | ***C*** |
|  |  | Injectable BPG unavailable near home |  |
|  |  | BPG stopped by a local physician after valvular intervention |  |
| Okello et al 2017 (49) | S1 | Physical capability barrier | ***U*** |
|  |  | Increasing age (71% of those of those <15 years compared to those >50 years |  |
|  | S2 | Pyschological capability barrier | ***C*** |
|  |  | No formal education |  |
|  | S3 | Physical capability barrier | ***Excluded*** |
|  |  | Presence of comorbidities such as the presence of stroke, atrial fibrillation |  |
| Anderson, et al., 2019 (44) | T1 | Social opportunity barrier | ***C*** |
|  |  | feeling dehumanized, Personally‐mediated racism and a lack of cultural safety |  |
|  | T2 | Physical opportunity barriers | ***U*** |
|  |  | Financial pressure, securing transport, meeting the costs of health care and prescription fees, long clinic wait, large number RHD patients to be cared for, patient mobility or residence change, lack of RHD registry, lack of age‐based approaches to service delivery esp. adolescents. lack of smooth pediatric to adult care transition |  |
|  | **T3** | **Reflective motivation barrier** | ***C*** |
|  |  | receiving rough handling during BPG injection, being perceived as different in school-based BPG injections |  |
|  | T4 | **Physical opportunity facilitator** | ***U*** |
|  |  | School-and home-based BPG delivery |  |
| Huck., et al., 2015 (8) | U1 | **Social opportunity facilitator** | ***U*** |
|  |  | support from family and friends, positive relationship with healthcare providers, appointment reminder by family |  |
|  | U2 | **Automatic motivation barrier** | ***U*** |
|  |  | BPG injection pain |  |
|  | U3 | Social opportunity barrier | ***C*** |
|  |  | Poor relationship with family, friends and healthcare providers |  |
|  | U4 | **Psychological capability barrier** | ***U*** |
|  |  | Lack of knowledge on RHD and BPG injection |  |
|  | U5 | **Physical opportunity barrier** | ***C*** |
|  |  | Treatment and transport costs, poor availability of BPG, clinics and healthcare providers, long clinic wait time |  |
|  | U6 | **Physical opportunity facilitator** | ***U*** |
|  |  | Appointment reminders like health clinic cards, receipts, medical documents; close residence to the clinic |  |
|  | U7 | **Reflective motivation facilitator** | ***C*** |
|  |  | Perceived worsening of RHD with missing BPG, personal motivation or responsibility for one's health |  |
| Nalubwama, et al., 2023(46) | V1 | Physical opportunity barrier | ***U*** |
|  |  | Long distance and cost of travel |  |
|  | V2 | Automatic motivation barrier | ***U*** |
|  |  | Painful BPG injections |  |
|  | V3 | Psychological capability barrier | ***C*** |
|  |  | Inadequate knowledge about RHD and BPG |  |
| Volti, et al., 2020 (51) | W1 | **Physical opportunity barrier** | ***C*** |
|  |  | Financial limitations, transportation cost/availability, cost of medication, lack of healthcare workers, and distance to higher-level facilities. |  |
|  | W2 | **Automatic motivation barrier** | ***C*** |
|  |  | Fear of injection |  |
|  | W3 | **Psychological capability barrier** | ***U*** |
|  |  | Misunderstanding: Health literacy: “I used to sell charcoal, we could go and buy it from far places, climb those Lorries, and I think that is where I got this disease from.” |  |
