# Supplementary material for: Barriers and facilitators of benzathine penicillin G adherence among rheumatic heart disease patients: a mixed methods systematic review using the COM-B (capability, opportunity, and motivation for behavior) model
Source: Syst Rev. 2024 Dec 3;13:297. doi: 10.1186/s13643-024-02691-1 (PMC11613468; doi:10.1186/s13643-024-02691-1)
Supplement: Supplementary file 4 — Additional file 4: Table S4: Data transformation. [file 13643_2024_2691_MOESM4_ESM.docx]

##### **Table s2: Data transformation**

| **Study** | **Design** | **Quantitative finding** | **Qualitatized findings** |
| --- | --- | --- | --- |
| Musoke, 2013 (59)    (Uganda) | Cohort | - The age range was 5 to 55 years, with a mean of 28.1 years (SD 12.2) and median of 28 years. - The majority were females, 78.9%. (F: 75 & M: 20). - Residence: near to two-third lives in urban 60% & rural 40% - Benzathine penicillin adherence: 44 (54%) adhered to the monthly benzathine penicillin prophylaxis, with adherence rates ≥ 80%; 38 (46%) patients were classified as non-adherent to the monthly benzathine penicillin, with rates less than 80%.The mean adherence level was 70.12% (SD 29.25) and the median level was 83.30%, with a range of 0-100%; 27 (33%) patients had extremely poor adherence levels of ≤ 60%. - The commonest reason for missing a dose was the painful benzathine penicillin injection (29%) followed by lack of transport money. - No BPG adherence difference between the RHD passport and the Self-report (p>0.05) - No association (p>0.05) found between adherence level and education status and residing near health facility. - The level of non-adherence was significantly high (46%). Residence in a town/city and having at least a secondary level of education was associated with better adherence, while the painful nature of the benzathine penicillin injections and lack of transport money to travel to the health centre were the main reasons for non-adherence among RHD patients. | - The median age was 28 years. - Majority of the partcipants were females. - Residence: near to two-third lives in urban and the rest lives in rural area which was related to lowere level of BPG adherence. - Poor BPG adherence level reported in the study area (54%: ≥ 80% doses). - Painful benzathine penicillin injection was reported as a main reason for missing injection followed by lack of transport money. - No BPG adherence difference between the RHD passport and the Self-report. - Age, sex, educational status, and clinical symptoms and BPG use experience were not related to BPG adherence were not related to BPG adherence. |
| Culliford-Semmens 2017 (43)  (New Zealand) | Cohort | - The majority were males, 53%. - The median age was 12 years. - BPG adherence was 92% (receiving≥80% doses). - RHD registry-based-BPG delivery was the facilitator for BPG adherence compared to primary health care penicillin delivery (p<0.005). | - Over half of the respondnets were males. - The median age was 12 years. - Most of the partcicipnta were adherent to BPG prophylaxis in the study area. - RHD registry-based-BPG delivery related to better BPG adherence compared to primary healthcare penicillin delivery. |
| Engelman, 2016 (40)  (Fiji) | Cohort | - Out of the total 494 patients, 54% were female and the median age was 14 years. - Adherence: 7% had adequate adherence. - Increasing age (OR 0.93 per year, 95% CI 0.87–0.99) and time since diagnosis ≥1.5 years (OR 0.53, 95% CI 0.37–0.79) to be inversely associated with any adherence. Urban residence (OR 3.36, 95% CI 1.54–7.36) were associated with adequate adherence, whereas time since diagnosis ≥1.5 years (OR 0.38, 95%CI 0.17–0.83) was inversely associated with adequate adherence. | 494 patients were included in the study.Overhalf of the repondnets were were female (54%) and the median age was 14 years.Adherence: very low level of the repondnets had adequate adherence (7%).Living in Urban area related to better adherence to BPG prophlaxis.Increasing age and time since diagnosis were related to inadeuate adherence to BPG prophlxis. |
| Balbaa, 2015 (56)    Egypt | Cross-sectional | - A total of 29 RHD patients and or parents/guardians were included. - About 65.5% adherent and 34.5% non-adherent (adherence was assessed at >75%). - Majority (68.5%) of adherent patient were knowledgeable compared to non-adherent about RHD(P=0.021) RHD knowledge, awareness of the effect of missing prophylaxis and family appointment reminders were facilitators for BPG adherence. - Majority of the adherent participants were aware of the consequences of missing prophylaxis doses compared to nonadherent (79% versus 40%, p= 0.005). - Intentional avoidance of BPG injection was observed in 90% of non-adherent patients (as compared to 31.6% of adherent patients, p = 0.005). - Prolonged wait clinic time were the barriers to BPG adherence. Clinic wait time was the most frequently reported deterrent for both groups. | - RHD patients and or parents/guardians were included in a study and over two-third (65.5%) of the participants were adherent. - Better understanding/knowledge of the disease (RHD) ehnanced adherence to BPG prophlyaxis - awareness of the effect of missing prophylaxis was related to better adherence to BPG prophylaxis. - family appointment reminders - Most of non-adherent RHD patients were intentionally avoiding thier BPG injection compared to the adherent patients. - prolonged wait clinic time was most report barrier for inadequate BPG prophylaxis adherence. |
| Arvind, 2021 (55)  India | Cross-sectional | - Form the total 420 patients, majority were male (65.2%), from rural (87.1%) areas, and belonged to lower socioeconomic strata (73.3%) with a mean age (±SD) 11.6 (±2.9) years]. - Many of the participants took BPG from the private clinic and had to walk far. - Most of the participants were adherent (92.2%) of the BPG doses. - Lack of knowledge regarding the importance of secondary prophylaxis was the most common cause for missing doses and others because of misinformation by the treating physician. - There was no associated factor in socioeconomic variables. - No difference in rural/urban areas, expenses and distance travelled regarding patient compliance. - The costs for transportation constituted 50% of the total costs, whereas the mean cost for drug procurement and drug administration constituted 30% and 22% of the total costs respectively. | - The mean age was 11.6 ±2.9years - The majority were male, from rural areas, and belonged to lower socioeconomic strata. - Most of the participants (92.2%) were adherent. - Many of the participants took BPG from the private clinic and had to walk far. - Lack of knowledge regarding the importance of secondary prophylaxis remain main reported barrier. - misinformation by the treating physician. - There was no difference in rural/urban areas, expenses and distance travelled regarding patient compliance. |
| Adal. 2022 (41)  (Ethiopia) | Cross-sectional | - 381 RHD patients participated with a mean age of the respondents was 26.45 ±10.5 years. - The median age of the study participants was 26.5 years and majority were females, 59.8% and most were from urban areas (80.1%). - BPG adherence was 70.9% (receive ≥80% doses). - Rural residency (AOR = 2.637, 95% CI: 1.068–6.513), living with > family members (AOR = 2.879, 1.282–6.465), living more than 30 km from health clinic (AOR = 3.247, 95% CI: 1.051–10.033), lack of penicillin (AOR = 6.772, 95% CI: 3.234–14.177) and fear of catching Covid-19 (AOR = 0.04, 95% CI: 0.014–0.114) were independently associated with risk factors for poor adherence. - Most of the participants were aware that bacteria are responsible for RHD and treatment with BPG prevents its recurrence and worsening. - Rural residence (p = 0.036), residing in a family of more than 5 members (p < 0.01), residence of more than 30-Km from a health facility (p < 0.041), lack of BPG in a follow up hospital, and fear of catching COVID 19 (P < 0.01) were the barriers to BPG adherence. - Reported reasons for missing medication were shortage of supply, fear of injection pain and poor awareness of prophylaxis. - Age, educational status, health insurance, hospital admission history and co-morbidities didn’t affect BPG adherence. | - The median age was 26.5 years. - The majority were females, 59.8% and most were from urban areas (80.1%) - Majority (70.9%) of the participants’ BPG prophylaxis adherence was adequate. - Adherence to BPG prophylaxis was enhanced by both awareness that bacteria are responsible for RHD and BPG prevents its recurrence and worsening. - Living in rural area was linked with inadequate adherence. - RHD patients residing in a family of more than 5 members and traveling from more than 30km were inadequately adherent to BPG prophylaxis. - Other barriers for BPG adherence were lack of BPG in a follow-up hospital, fear of catching COVID-19, fear of injection pain and poor awareness of prophylaxis. |
| Zewde, 2022 (52)  Ethiopia | Cross-sectional | - 385 RHD patients included and mostly females, 71.7% with a mean age was 31 years. - The majority, 79.6%, were knowledgeable about RHD's association with sore throat. - Three-fourths, 75.6%, believed that BPG prevents tonsillitis. - One-third,32.2%, believed that BPG cures RHD and close to one-tenth, 7.9%, also believed that BPG prevents RHD progression. - BPG adherence was 77.9% (receive ≥80% doses). BPG unavailability, older age, missing appointments, and BPG injection refusal by healthcare provider were the barriers to BPG adherence. - BPG injection was not related to BPG adherence. - Fear of injection was not related to any level of adherence | - 385 RHD patients included and mostly females, 71.7% with a mean age was 31 years. - Majority of the RHD patient had adequate BPG adherence, 77.9%. - Majority of the respondents were knowledgeable about RHD's that could facilitated for better adherence to BPG prophylaxis. - Over three-fourths, believed that BPG prevents recurrence. - One-third believed that BPG cures RHD. - Close to one-tenth, believed that BPG prevents RHD progression. - BPG unavailability, older age, missing appointments, and BPG injection refusal by a healthcare provider, forgetting BPG schedule were linked with poor adherence. |
| Adem, 2020 (53)  (Ethiopia) | Cross-sectional | - The average age was 24 years. The mean age was 24 ±11 (6–65) years. The majority were females, 70.4% of the total participants(n=253). - Good adherence was 63% (receiving≥80% doses). Rural residence (AOR 12.6 [95% CI 2.5–63], P=0.016; p= 0.003), long distance from a health facility(>30-kms) (AOR 6.8 [95% CI 1.9–24.4], p=0.031), duration of BPG injection (>5 years) (p= 0.061), mild symptoms (p= 0.016) were the barriers to BPG adherence. - Leading barriers to good adherence were long distance from the treatment setting (56.9%), followed by lack of money (38%).   Sex, age, family size, duration of RHD illness, and hospitalization history were not related to BPG adherence. | - Majority of the participants were females with average age of 24 years. - Good adherence was reported in two third of the participants (63%). - Adherence to BPG prophylaxis was affected by living in rural area, coming from long distance from a health facility(>30-kms), duration of BPG injection (>5 years), lack of money and having a mild symptom |
| Alkan, 2022 (54)  (Turkey) | Cross-sectional | - 43 participants from paediatric cardiology with a mean age of 16.2 ± 2.83 years included. - More than two-thirds, 31 (72%) of the participant patients were regularly following their secondary prophylaxis. - Fear of syringes and forgetting to get a prescription and/or take the drug when the time comes (p<0.05) negatively affects adherence. - Forgetting to get a prescription and/or take the drug when the time comes was statistically higher in the non-adherent group (p = 0.009). - However, age, gender, living in rural areas, educational status of mother and fathers, whether the patients received enough information, lifestyle, fear of developing adverse effects, fear of addiction, lack of health insurance, or difficulties in reaching the drug or hospital did not have relevant influence or significance on adherence nor did psychosocial factors. | - 43 participants with a mean age was 16.2 ± 2.83 years were participanted in the study. - Adequate adherence reported in majority of RHD patients(72%). - Inadequate adherence was related to fear of syringes/injection, forgetting to get a prescription and/or take the drug when the time comes. |
| Engelman, 2017 (57)  (Fiji) | Cross-sectional | - A total of 101 RHD patients with the median age of 17.2 years (14.6-19.4). - The level of good adequate adherence to secondary prophylaxis was sixty percent. - Travel to clinics other than their local health centres to receive injection. Unavailability and logistic reasons of proximity of the clinic to family, school, or employment were main reasons for inadequate adherence to BPG prophylaxis. In addition, the most common reasons mentioned for missing injections were lack of understanding that injections were required, feeling well and healthy, and transport unavailability. More than a quarter (28%) of the participants mentioned that pain during injection was the main reason for missing their injections. Reminder strategies, particularly phone-based reminders, were considered helpful by most of the participants. | - 101 RHD patients with a median age of 17.2 years - A good level adherence was reported in two third of the study participants (60%). - Better adherence was observed in RHD patient who used reminder strategies, particularly phone-based reminders. - Inadequate adherence was related to distance travelled to receive the injection. - Their main reasons are unavailability and logistic reasons of proximity of the clinic to family, school, or employment. - Lack of understanding that injections were needed was also linked to inadequate adherence. - Other factors reported were feeling well and healthy, transport unavailability, lack of drug supply at the clinic and pain during injection. |
| Edwards 2021 (9)  (Sudan) | Cross-sectional | - 397 RHD patients in Khartoum included in this study, most (75%) of which were female with a median age was 40 years. - Only thirty two percent had the expected good adherence to BPG prophylaxis. - Younger age, shorter health facility wait time, perceived adequacy of healthcare facility staffing, treatment costs, and patients’ understanding of RHD disease are important and significant factors in enhancing adherence to BPG prophylaxis. For every year increase in subject age, there was a 2.7% decrease in odds of adherence (Odds Ratio (OR) = 0.973; 95% CI 0.952–0.995). For every minute increase in wait time at the subject’s RHD care facility, there was a 0.3% decrease in odds of adherence (OR = 0.997, 95% CI 0.994–0.999). Study participants who felt that there was adequate staffing at their healthcare facility had over a two-fold increase in likelihood of BPG adherence (OR = 3.472, 95% CI 1.475–8.172). - Household monthly income, educational level, and rural or urban residence also influenced adherence to some extent although they were not observed to be strong factors. - Patients who reported a lack of understanding of RHD as their primary barrier to appropriate treatment were 78% less likely to be adherent to BPG (OR = 0.319, 95% CI 0.164–0.619). - Interestingly, for every 1 SDG (~ 0.15 USD in 2017) increase in treatment cost, there was a 1.5% increase in odds of adherence (OR = 1.015; 95% CI 1.002–1.028). | - 397 RHD patients, over three fourth of the patients, were females with a median age were forty years. - only thirty-two percent had good adherence to BPG prophylaxis. - Younger age, shorter wait time, perceived adequacy of healthcare facility staffing, and treatment costs were considered factors related to enhanced adherence. - Patients with better understanding of RHD disease were related to better adherence. - Perception of improved symptoms with treatment [BPG prophylaxis injection] linked to better adherence. - Better household monthly income, educational level, and residence mildly influenced adherence. |
| Nemani, 2018 (7)  (India) | Cross-sectional | - From 500 RHD patients included (261 compliant (52.2%) and 239 noncompliant patients). - Average age of presentation was 29 ± 13 years with females outnumbering the males. - Non-compliance with the secondary prophylaxis of BPG was more common among male (p = 0.003), low socioeconomic class (p= 0.009), uneducated (P = 0.0018), and the rural population (p= 0.025) while those with previous history of rheumatic fever (RF) were found to be more compliant (p= 0.04). - Recurrences of RF were more common in those not on regular prophylaxis (p= 0.011). - Those with a history of rheumatic fever recurrence were also more likely to be more adherent to some extent. However, age, rural residence religion, acute rheumatic fever at presentation and severity of RHD didn’t have a significant relation with adherence rate. - The most cited reasons for non-compliance were insufficient counselling (like never told, stopped after the first dose, following surgery) by sixty-one percent and told but neglected (like feeling better, pain, financial and transport problems, shortage of medicines and allergy in few cases) by thirty-nine percent. | - Over half (52.2%) of the participants were compliant with BPG prophylaxis. - History of RF recurrence was reported as enahncer of adherence to BPG prophylaxis. - Noncompliance to BPG prophylaxis was more common among males, low socioeconomic status, and uneducated RHD patients. - Insufficient counseling by sixty-one percent and told but neglected (like feeling better, pain, financial and transport problems, shortage of medicines and allergy). - The most common reason cited for noncompliance was the absence of proper counselling followed by a sense of well‑being, injection site pain and financial constraints. |
| Sial, 2018  (33)  Pakistan | Cross-sectional | - Close to seventy percent (68.8%) of the participants were females, and their mean age was 34.09. - Almost three-fourths (73.5%) of the patients were adherent to their BPG secondary prophylaxis. - Patients presenting to the emergency department had a lower adherence rate (sixty percent of patients) as compared to those at the regular outpatient department. - Patients above 30 years had lower level of BPG prophylaxis adherence. - Adherence level has also related to the type of heart valve lesion in RHD with adherence observed among seventeen percent of aortic stenosis, and sixty percent of aortic regurgitation compared to seventy-nine and seventy percents of mitral stenosis and mitral regurgitation, respectively | - close to seventy percent of the participants were females, and their mean age was 34.1 years. - almost three-fourths of the patients were adherent to BPG prophylaxis. - patients presenting to the emergency department had a lower adherence rate. - age over 30 years linked to inadequate adherence. - Presence of heart valve lesion was associated with inadequate level of adherence to BPG prophylaxis. |
| Mekonen, 2020 (34)  (Ethiopia) | Cross-sectional | - 145 RHD patients, majority were females, 75.9% and majority were in their thirties, were included. - Half of RHD patients didn’t know why they took BPG, 48.3%. - BPG adherence was 80.6% (receive ≥80% doses). - History of no hospitalization (p =0.006) or one time hospitalization (p=0.007) was the facilitator for BPG adherence. - Skipping BPG injection (p=0.000) after a missed BPG dose until the next appointment was identified barrier to BPG adherence. - Education, religion, duration of BPG prophylaxis, wait time in BPG injection health facility, knowledge on BPG injection, and reasons for missed/late BPG doses were not related to BPG adherence. | - The study included 145 RHD patients and over three fourth of them were females with a mean age was 30.12± 9.62years. - Most of the participants were in better BPG adherence level, 80.6%. - Adequate level of adherence to BPG prophylaxis was linked with **h**istory of no hospitalization or one time hospitalization. - Half of RHD patients didn’t know why they took BPG which was also linked to inadequate adherence to prophylaxis. - skipping BPG injection after a missed BPG dose until the next appointment. |
| Muhammed, 2020 (47)  (Ethiopia) | Cross-sectional | - 135 were paediatric age group (age 5-16 years) and 106 were adult age group as the overall age ranges from 5-68 years. - Majority from rural part 172 while urban account 69 55.2% were leveled as good adherence ((≥80% doses). - Lack of money, distance from hospital, fear of medication side effects, painful injection, lack of knowledge about the disease and prevention were associated with lower level of BPG adherence. | - 241 participants with age ranges between 5 to 68 years were included. - Majority were from rural part - Only 55.2% RHD patients had good adherence - The inadequate adherence in this study area were due to lack of money, distance from hospital, fear of medication side effects, painful injection, lack of knowledge about the disease and prevention. |
| Awan,2021  (48)  Pakistan | Cross-sectional | - 195 patients, most were female, 66.7%. - The mean age was 32.25 years. - BPG Adherence: 73.6% - Reasons for inadequate adherence for BPG were painful injections, mostly allergic reaction, no nearby health facility, injection not available at nearby health facility, friends/family advised otherwise, felt sick and unable to take injection, pregnancy, lactation, financial constraints, and unspecified reasons. | - Painful injections (most common reason), experience allergic reaction, lack of access to healthcare in nearby, injection unavailability at nearby health facility, friends/family advised otherwise, felt sick and unable to take injections, and financial constraints identified reason for inadeuate adherence to BPG prophlaxis. |
